# Relating brain function to cognitive-visuomotor integration performance in working-aged adults with persisting concussion symptoms

**DOI:** 10.3389/fnhum.2026.1772037

**Published:** 2026-04-28

**Authors:** Miracle E. Ozzoude, Diana J. Gorbet, Alison K. Macpherson, Lauren E. Sergio

**Affiliations:** 1 Institute for Neuroscience and Cardiovascular Research, University of Edinburgh, Edinburgh, United Kingdom; 2 School of Kinesiology and Health Science, Faculty of Health, York University, Toronto, ON, Canada; 3 Centre for Integrative and Applied Neuroscience, York University, Toronto, ON, Canada

**Keywords:** brain networks, cognitive-motor integration, concussion, cortex, eye-hand coordination

## Abstract

**Introduction:**

In everyday life we interact with our environment in an indirect way, where there is a mapping between the viewed goal of our action and the required movement (e.g., using a computer mouse). Such tasks require cognitive- motor integration (CMI), where rules dictate the relationship between perception and action. Previous research with primarily young adult male athletes has demonstrated that the underlying movement and cognitive control networks that rely on intact frontal, parietal, and subcortical brain region connectivity may be compromised following concussion, resulting in an impaired ability to engage in complex movements. Here we investigate whether such relationships also exist in working-aged adults with persistent post-concussion symptoms (PPCS).

**Methods:**

Twenty-two individuals (17 females) performed two visuomotor tasks: one requiring direct (standard) interaction with visual targets, and one comprising a plane-change and feedback reversal (non-standard interaction) between viewed target and required hand motion (CMI). PPCS and dizziness were related to brain network function via resting state functional connectivity (RSFC) in six networks, structural integrity via cortical thickness in CMI-related brain regions, and white matter tract integrity via diffusion tensor imaging.

**Results:**

We observed that lower cortical thickness in the inferior and superior parietal cortices were associated with dizziness and impaired non-standard visuomotor performance, respectively. Furthermore, increased PPCS severity was associated with hyperconnectivity within the visual, sensorimotor control, frontoparietal control, and dorsal attention networks, while hyperconnectivity within the salience ventral attention network was associated with better non-standard visuomotor performance. Lastly, we found that lower white matter tract integrity in several long associative, projection, and commissural tracts were associated with poor cognitive-motor integration performance, PPCS severity, and dizziness.

**Discussion:**

These preliminary findings characterize the impact of PPCS on structure and function underlying impaired visuomotor performance.

## 1 Introduction

Concussion is a form of mild traumatic brain injury (mTBI) that results in a clinical syndrome due to biomechanical forces acting on the brain, produces a constellation of symptoms, and can arise from a variety of mechanisms (Dimou and Lagopoulos, 2014; McCrory et al., 2017; Pardini et al., 2010; Prigatano and Gale, 2011). Approximately 10–30% of individuals experience cognitive, physical, and somatic symptoms that persist over an extended period of time (i.e., months, years, or permanently), an occurrence termed persisting post-concussion symptoms (PPCS; McCrory et al., 2017; Prigatano and Gale, 2011; Toledo et al., 2012). Although there is a deliberation over the definition of PPCS, the diagnosis is defined according to the International Classification of Diseases as a mild neurocognitive disorder with a subjective experience of a deterioration from a previous level of cognitive functioning, accompanied by objective evidence of impairment in performance on one or more cognitive domains due to TBI (World Health Organisation, 2019). Understudied aspects of PPCS include risk factors associated with increased likelihood of PPCS, such as previous history of concussion, pre-existing psychological and mood disorders, age, and sex/gender (Fino et al., 2017; Sharp and Jenkins, 2015; Broshek et al., 2005; Brown et al., 2015; King, 2014; Varriano et al., 2018).

Structural, or anatomical, magnetic resonance imaging (sMRI) detects declines in gray matter morphology as an indicator of atrophy resulting from focal and/or global neuronal loss (Symms, 2004). Although there are no visible concussion-related structural changes observable in conventional neuroimaging methods such as computerized tomography and sMRI (Klein et al., 2019), many studies have demonstrated alterations in gray matter morphometry using advanced neuroimaging techniques (Burrowes et al., 2020; Hurtubise et al., 2022; Mavroudis et al., 2022; Niu et al., 2020; Mills et al., 2020; Wang et al., 2015). Further, biomechanical forces such as mechanical shearing and tensile strain associated with concussion initiate a neurometabolic cascade and cytoskeletal breakdown from damage to axons. These pathophysiologic processes may lead to disrupted axonal transport, demyelination, and slowed neurotransmission, resulting in the loss of white matter microstructural integrity (Barkhoudarian et al., 2011; Choe, 2016; Giza and Hovda, 2014), changes that can be detected using diffusion tensor imaging (DTI, Taylor et al., 2003). Multiple studies have demonstrated that specific white mater tracts are particularly vulnerable to traumatic damage (Gumus et al., 2021, 2022; Rabinowitz et al., 2014). In terms of movement control, Farbota et al. (2012) observed white matter integrity changes that were associated with both slower and faster visuomotor speed. In addition to microstructural changes, functional connectivity changes have been documented post- concussion. Similarly, alterations in resting state functional connectivity (RSFC) have been observed after concussion in a number of networks, including the default mode (DMN), salience ventral attention (SVAN), and the frontoparietal control (FPCN) networks (Gumus et al., 2021; Hayes et al., 2016; Rabinowitz et al., 2014; Rosenthal et al., 2018; Sharp et al., 2014; So et al., 2023). The DMN is essentially involved in internally-directed cognition, such as metallization (Spreng et al., 2009), autobiographic memory (Andrews-Hanna et al., 2014a), and emotional processing (Andrews-Hanna et al., 2014b). However, during the anticipation of and the responding to tasks that require attention to the external environment, this network is inhibited, allowing the brain to immediately switch to task-positive networks (e.g., SVAN and FPCN) (Dixon et al., 2018; Menon and Uddin, 2010). As such, both increases and decreases in RSFC have been reported in these networks and such changes are correlated with cognitive and behavioral impairments, and PPCS severity (Goswami et al., 2016; Gumus et al., 2021; Mayer et al., 2015; Wong et al., 2023; Kasahara et al., 2011; Stevens et al., 2012; Churchill et al., 2021). Several studies have suggested that increased RSFC in the presence of abnormal structural integrity may reflect compensatory recruitment of additional neural resources to sustain cognitive processing (Rosenthal et al., 2018; Sharp et al., 2014; So et al., 2023; Bharath et al., 2015) found initial increased DMN RSFC spread to other brain networks after 3 months, and their patients had neurocognitive test scores and RSFC that were comparable with healthy controls after 6 month post-injury. Conversely, increased RSFC may represent the desynchronization of neurons due to brain injury, resulting in a need for hyperconnectivity to achieve the same neural signal (Meier et al., 2017; Schultz et al., 2017).

With respect to complex motor task execution, while many of our activities of daily living involve simple direct interactions in which the guiding visual information is also the goal of the movement, an increasingly technology-driven world has introduced many situations which require indirect interactions. An example of a direct standard interaction is looking at and then reaching to grab a cup of tea while an example of an indirect non-standard interaction is driving a car, where the limb motions are decoupled from the motion of the vehicle being operated. Successful completion of the non-standard interaction requires the integration of cognitive and motor skills. Moreover, cognitive-motor integration (CMI) explains how our brains plan and execute movements when there is a complex relationship between sensory input and motor output, especially in reaching movements when the hand and eyes are decoupled. This is because CMI is essential during these incongruent hand-eye coordination tasks, where rules dictate the association between perception and action (Sergio et al., 2020; Wise et al., 1996; Bock, 2005; Redding and Wallace, 1996). The performance of both standard and CMI tasks involves the activation of multiple cortico-subcortical brain networks, especially the frontoparietal-cerebellar and attention networks (Dixon et al., 2018; Ohashi et al., 2018), in addition to recruiting white matter tracts along these networks (Brandes-Aitken et al., 2019). Moreover, there are differences in motor performance – with CMI performance being worse on a variety of kinematic measures - as well as the underlying patterns of network activation between both tasks (Gorbet and Sergio, 2009; Sergio et al., 2020; Messier and Kalaska, 1997; Wise et al., 1996), likely reflecting the extra neural processes required for such multi-domain (i.e., sensory, cognitive, motor) integration. Recent research has demonstrated alterations in both RSFC of these aforementioned networks and white matter integrity due to aging, concussion, and neurodegeneration (Hawkins et al., 2015; Hurtubise et al., 2020, 2022; Rogojin et al., 2022, 2023). Notably, several studies have reported sex-related differences in CMI performance and brain network activity controlling CMI (Gorbet et al., 2010; Gorbet and Sergio, 2007; Gorbet and Staines, 2011; Rogojin et al., 2019; Sergio et al., 2020; Smeha et al., 2022). While these findings may provide possible insight into the neural correlates of CMI performance in sport-related concussion in young athletic adults and sex differences in these correlates, it is important to expand this work to other concussion mechanisms and working-aged adults.

To this end, given prior research demonstrating that CMI performance is affected by sport and videogame experience (Gorbet and Sergio, 2018; Granek et al., 2010) in addition to brain health declines resulting from neurodegenerative disease (Hawkins et al., 2015; Rogojin et al., 2019, 2022, 2023) and concussion (Hurtubise et al., 2016; Hurtubise et al., 2020, 2022; Smeha et al., 2022), the main objective of this pilot study was to characterize the impact of PPCS on the functional and structural neural underpinnings of visuomotor performance in working-aged adults. Examining this demographic is imperative because previous studies have mainly focused on sport-related concussion in children and adolescents, university-aged athletes, and elite athletes. Therefore, the current study provides a novel approach in addressing the aforementioned relationship in working-aged adults with various concussion mechanisms. Based on our previous findings, we hypothesized that participants with PPCS would demonstrate behavioral deficits on visuomotor tasks, especially in conditions involving CMI, and that these deficits would be associated with alterations in RSFC and reduced white and cortical gray matter integrity. Importantly, we hypothesized that these associations would be observed in networks implicated in visuomotor control namely the visual, dorsal and ventral, sensorimotor control, and frontoparietal control networks, as well as several white matter tracts and cortical regions subserving these networks. Lastly, we predicted that alterations in brain functional connectivity and structural integrity would be related to PPCS severity.

## 2 Materials and methods

### 2.1 Participants

Twenty-two working age participants between the ages of 30 and 65 years were included in the current study (47.23 ± 9.26; 5 males). While we recognize that the term “working age” is between the ages of 16–65 years old, we reduced this range given the influence of age on cognition and motor control and focused on the age range for which we have the least available data. This is because our prior work has focused on youth, young adults, and post- retirement seniors (Gorbet and Sergio, 2007, 2016; Hurtubise et al., 2020; Rogojin et al., 2019). In addition to recruiting participants with PPCS (>3 months post-incident), exclusion criteria included uncorrected visual impairments, a history of stroke, epilepsy/seizures, active vestibular or neurodegenerative disorder(s) with the etiology other than concussion (e.g., Meniere’s disease or Parkinson’s disease), acute psychiatric disorder(s), diagnosis of dementia or mild cognitive impairment, inability to provide informed consent, and inability to speak and understand English or French. A diagnosis of concussion/PPCS relied either on the accuracy of the participant’s physician, referring clinic, or the date and mechanism of injury was recalled. All participants completed a health questionnaire which included mechanism of each concussion, number of previous concussions, and time since last concussion. None of the participants had gross morphological abnormalities upon examination of MR images.

The study protocol was approved by the Human Participants Review Sub-Committee of York University’s Ethics Review Board, and all participants provided written informed consent.

### 2.2 Measures

**Figure 1:**
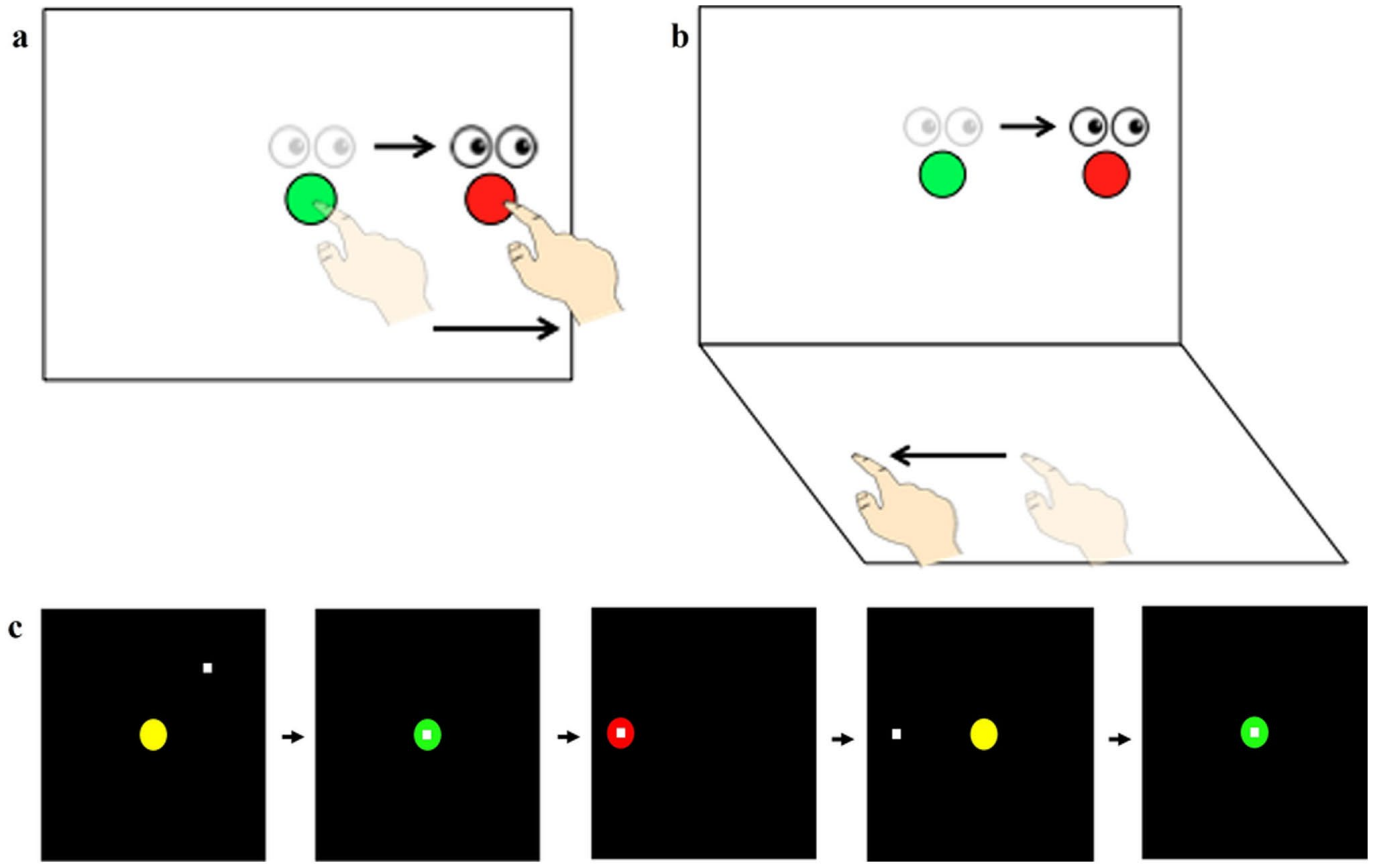
**(a)** Schematic drawing of the visuomotor transformation tasks. Lighter eye and hand symbols denote the starting position for each trial (green central target). Darker eye and hand symbols denote the instructed eye and hand movements for each task. Red circles denote the peripheral (reach) target, presented randomly in one of four locations (left, up, right, or down relative to the central target). The direct interaction/standard condition requires standard mapping, where participants slide their finger on a touch screen to move a cursor from a central target to one of four peripheral targets. **(b)** The non-standard condition is a cognitive-motor integration (CMI) condition, where targets are either spatially dissociated from the plane of hand motion (plane-change) and have a 180° feedback reversal (plane-change + feedback reversal, PC + FR). **(c)** Sequence of events during one trial of the visuomotor task. The central (home) target is where all trials begin. Once the participant moves the cursor (white square) into the central target, the target changes from yellow to green to signify a movement preparation period. After 2,000 milliseconds (ms), a red peripheral target appears in one of four directions (up, down, left or right of the center) and serves as the “go” signal. Once the peripheral target is acquired and held for 500 ms it disappears, signaling the end of the trial. After an inter-trial interval of 2,000 ms, the central yellow target reappears, and the participant moves back to the central target to start the next trial. Adapted from Rogojin et al. (2022).

1. Persistent Post-Concussion Syndrome (PPCS) was assessed using the Rivermead Post-Concussion Symptoms Questionnaire (RPQ) (Eyres et al., 2005; King et al., 1995; Potter et al., 2006). RPQ is a 16-item self-report standardized questionnaire that records the presence and severity of PPCS. The 5-point ordinal scale ranges from 0 (not experienced at all) to 4 (a severe problem) with a higher score reflecting greater severity of PPCS. The RPQ is made of two groups. The first group consists of the first three items (RPQ-3: headaches, dizziness, nausea) and are associated with the early physical symptom clusters of PPCS. The second group comprises of the next 13 items (RPQ-13: noise sensitivity, sleep disturbance, fatigue, irritability, depression, frustration, forgetfulness, poor concentration, taking longer to think, blurred vision, light sensitivity, double vision, restlessness) and are associated with later psychological and cognitive symptom clusters of PPCS although the items from RPQ-3 might also be present. Note that for this scale, a score of 1 reflects “no more a problem,” meaning the symptom was present but is no longer endorsed as a symptom by the participant. 2. The impact of dizziness-related disability on quality of life was assessed using the Dizziness Handicap Inventory (DHI). DHI is a 25-item self-reported questionnaire assessing the physical, functional, and emotional components of vestibular dysfunction (Jacobson and Newman, 1990; Mutlu and Serbetcioglu, 2013). The 3-point ordinal scale ranges from 0 (No) to 4 (Always) where a high score indicates an increased level of self- perceived handicap of vestibular dysfunction. The total score was 100 divided into three domains consisting of physical (24 items), functional (40 items), and emotional (36 items). 3. Behavioral assessment task: A detailed description of our visuomotor assessment has been previously published (Hurtubise et al., 2022). Briefly, all participants completed two visuomotor transformation tasks that were delivered using custom-written software. These tasks were separated into one standard (direct) condition (target viewed and finger motion are spatially coupled) (Figure 1a) and one non-standard (CMI) condition (target viewed and hand motion are spatially decoupled twice) (Figure 1b). In all conditions, participants were instructed to slide the index finger of their dominant hand along the touch screen (either on a vertical touchscreen using an ASUS™ touchscreen tablet or on a horizontal touchpad using an external Keytech™ USB-touchpad that was positioned perpendicular to the ASUS tablet depending on the condition) in order to displace the cursor from a central target to one of four peripheral targets (up, down, left, right). The targets for the finger motions were presented on the vertical touchscreen in both conditions. In the standard (direct) mapping task, the spatial locations of the visual target and the required hand movement were the same (Figure 1a), i.e., participants both looked at and moved on the vertical touchscreen, thereby directly interacting with the targets. For the non-standard (CMI) mapping task, the finger movement was made on a different plane and in the opposite direction [plane-change + feedback reversal (PC + FR); Figure 1b] relative to the spatial target location. Importantly, the PC + FR condition required the participants to look at the vertical touchscreen while manipulating a cursor using their finger on the horizontal touchpad requiring implicit sensorimotor recalibration. The feedback was rotated 180°, i.e., in order to move the cursor to the left, the participant must slide their finger right, requiring explicit strategic control and movements of the eyes and hand to be made in opposite directions.

All conditions were randomized. The red peripheral targets were located 55 millimeters (mm) from the central target. The finger motion and trial timings consisted of: (1) a central yellow target with a diameter of 7.5 mm appearing on the center of the vertical touchscreen, (2) participants moving a white cursor to the central yellow target and changing its color to green once the cursor has entered the central target, (3) after a delay period of 2,000 milliseconds (ms), a red peripheral target appearing and the central target disappearing, indicating the “go” signal for initiation of a movement, (4) participants were told to look toward the visual target on the vertical touchscreen and slide their finger along the touchscreen or touchpad to direct the cursor toward the cued peripheral target (up, down, left, or right), (5) once the peripheral target has been reached and the participant has held that position for 500 ms, the peripheral target disappears, signaling the end of the trial, and (6) after a delay of 2,000 ms, the central yellow target reappeared, signaling the participant to return to the center for the next trial (Figure 1c). In all conditions, participants were instructed to move as quickly and accurately as possible. Each participant completed 20 trials per condition, i.e., 5 trials for each peripheral target.

### 2.3 Behavioral data processing

Kinematic measures, including timing, finger position, and error data were recorded for each trial and converted into a MATLAB readable format using a custom written C++ application. Unsuccessful trials (error data) were detected by the data collection software and resulted in trial termination if the finger left the home target too early (<2,000 ms), reaction time (RT) was too short (<150 ms), RT was too long (>8,000 ms), or total movement time was too long (>10,000 ms). Trials in which the first ballistic movement exited the boundaries of the center target in the wrong direction (greater than 45° from a straight line to target) were coded as direction reversal (DR) errors and analyzed as separate variables from the correct trials. A custom-written analysis program (Matlab, Mathworks, Inc., United States) was used to analyze the data from the collection program. Velocity profiles were computed for each successful trial and displayed alongside a Cartesian plot illustrating finger position data and target locations using a custom analysis program. The movement onsets and ballistic movement offsets (the initial movement prior to path corrections) were scored at 10% peak velocity, while total movement offsets were scored as the final 10% peak velocity point once the finger position plateaued within the peripheral target. In situations where the initial movement successfully brought the finger to the peripheral target, the ballistic and total movement offsets were equivalent. These movement profiles for each trial were verified by visual inspection and corrections were performed when necessary. The scored data were processed to compute 7 different movement timing, accuracy, and precision measures described below. Individual trials which exceeded 2 standard deviations (SD) from the participant’s mean for any of the outcome measures were eliminated prior to the calculation of outcomes.

The kinematic outcome measures were as follows: (1) Reaction time (RT), the time interval between the central target disappearance and movement onset measured in ms; (2) Full movement time (MTf), the time between movement onset and offset measured in ms; (3) Peak velocity (PV), the maximum velocity obtained during the ballistic movement measured in mm/ms, and used to calculate the 10% threshold used for determining movement onsets and offsets for each trial. (4) Path length (PL), the total distance (resultant of the x and y trajectories) traveled between movement onset and offset, measured in mm, calculated as both the full path length (PLf, start to final offset) as well as the ballistic trajectory (PLb, start to initial movement offset); (5) Absolute error (AE, end-point accuracy), the average distance from the individual movement endpoints (∑ x/n, ∑ y/n) to the actual target location, in mm; (6) Variable error (VE, end-point precision), the distance between the individual movement endpoints (σ2) from their mean movement, measured in mm; and (7) The percent direction reversal errors (%DR, only applicable in the PC + FR condition), the percentage of total trials that constituted a deviation of greater than ±45° from the direct line between the center of the central and peripheral targets. The procedure for combining some of the kinematic measures into composite scores to decrease the number of comparisons made in the data analysis was previously described (Rogojin et al., 2019). Briefly, all kinematic measures were standardized using z-scores and the composite scores were then created using simple averaging. A “timing score” was created as a composite of RT, MTf, and inversed PV (PV z-score * -1), and a “trajectory score” was a composite of PLf, AE, and VE. The timing and trajectory scores had a good internal consistency, as indicated by Cronbach’s alpha values of 0.760 and 0.447, respectively. The timing and trajectory composite scores, RT, PLf, and %DR were then used for statistical analysis.

### 2.4 Magnetic resonance imaging data acquisition

MRI data were acquired using a 3 Tesla (3 T) Siemens PrismaFit scanner at York University. Participants received a T1-weighted anatomical scan using a sagittal volumetric magnetization-prepared rapid gradient echo (MP-RAGE) sequence. The MP-RAGE consisted of the following acquisition parameters: 192 sagittal slices (slice thickness of 1 mm, with no gap), field of view (FOV) of 256 × 256 mm, matrix size of 256 × 256 resulting in a voxel resolution of 1 × 1 × 1 mm^3^, echo time (TE) = 2.26 ms, repetition time (TR) = 2,300 ms, flip angle = 8o. For assessing white matter (WM) integrity, whole-brain diffusion-weighted images (DWIs) were acquired with 64 directions using diffusion-weighted spin-echo single-shot echo planar imaging (EPI). The diffusion tensor imaging (DTI) sequence used the following acquisition parameters: 60 axial slices (slice thickness of 2.6 mm, with no gap), FOV of 220 × 220 mm, matrix size of 146 × 146 resulting in a voxel resolution of 1.5 × 1.5 × 2.6 mm^3^, TE = 84.0 ms, TR = 2,600 ms, *b*-value = 1,000 s/mm^2^ (including one volume with no diffusion gradient, *b* = 0 s/mm^2^). Additionally, we acquired two reversed phase-encoded DWIs (60 slices, voxel resolution = 1.5 × 1.5 × 2.6 mm^3^, TE = 84.0 ms, TR = 2,600 ms, *b*-value = 1,000 s/mm^2^, b = 0 s/mm^2^) corresponding to anterior–posterior/blip-up and posterior–anterior/blip-down, respectively. The rsfMRI was acquired with multi-band accelerator factor 4 and multi-echo EPI sequence sensitive to BOLD contrast. Participants were asked to lie in a scanner with their eyes open and fixate on a white cross with a black background for approximately 12 min during which the functional sequence with the following parameters was acquired: 52 axial slices (slice thickness of 3 mm, no gap), FOV of 240 × 240 mm, matrix size of 80 × 80 resulting in voxel resolution of 3.0 × 3.0 × 3.0 mm^3^, TR = 961 ms, and echo times (TEs) = 12.40, 30.15, 47.90 ms, flip angle = 50°. Each TR resulted in the acquisition of 3 volumes, one for each TE. The time-series for each TE were separately converted to nifti (nii) format resulting in one nii image per echo time. One resting functional run consisting of 2,274 images (758 per echo) was acquired for each participant.

#### 2.4.1 MRI preprocessing

**Figure 2:**
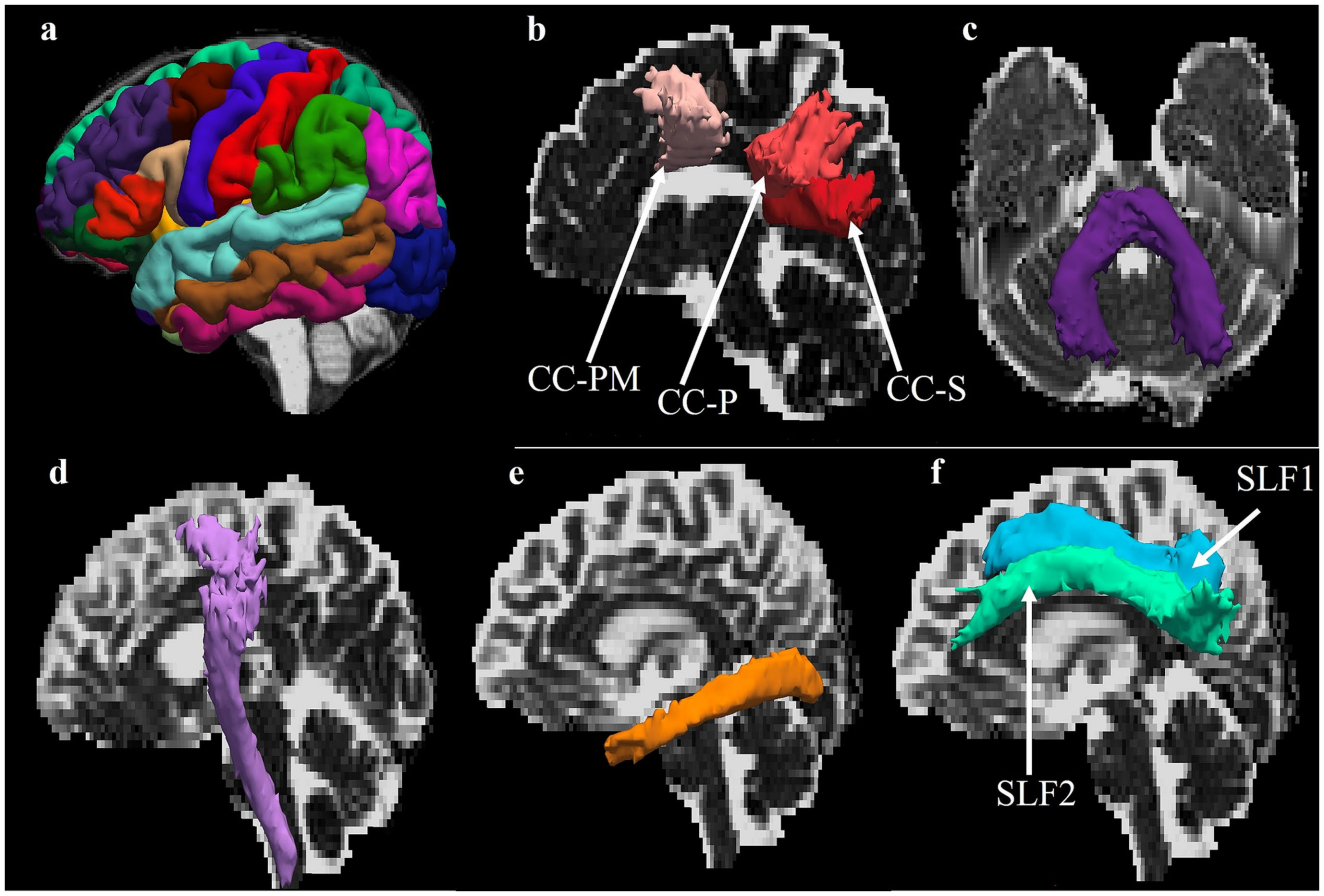
**(a)** 3D depiction of Desikan-Killiany atlas obtained from FreeSurfer parcellation of cortical regions. **(b–f)** White matter tracts of interest obtained from Tracula constrained by Underlying Anatomy (TRACULA), shown in **(b)** CC—PM, corpus callosum–premotor; CC—P, corpus callosum—parietal; CC-S, corpus callosum—splenium; **(c)** MCP, middle cerebellar peduncle; **(d)** CST, corticospinal tract; **(e)** ILF, inferior longitudinal fasciculus; **(f)** SLF, superior longitudinal fasciculus 1, superior longitudinal fasciculus 2.

1. Structural data: All anatomical scans was processed using FreeSurfer 6.0 (“recon-all”) pipeline with T1-weighted MR as input (Fischl, 2012). Briefly, the standard reconstruction steps include intensity correction, Talairach transformation, intensity normalization, skull stripping, subcortical segmentation, and cortical parcellation. Skull-stripping was performed on the Talairach transformed and intensity corrected and normalized image using a deformable template model. Voxels were then classified as either white matter, gray matter, or cerebrospinal fluid based on intensity values. Next, the segmentation of subcortical structures and generation of the cortical surface, followed by the classification of tissue intensities between the white and gray matters (referred to as white surface) and between the gray matter and cerebral spinal fluid (referred to as pial surface). All surfaces were constructed in the individual anatomical space. The surfaces were inflated into a sphere and registered to the FreeSurfer template sphere (fsaverage). The non-linear surface-based registration allowed for more accurate alignment of the gyri and sulci landmarks. A cortical parcellation of the template was then mapped back onto the individual participant and adjusted for small variations. The cortical parcellation was founded on the Desikan-Killiany atlas, a gyral-based atlas established using 40 participants (Desikan et al., 2006; Figure 2). Cortical thickness was calculated as the distance between the gray matter and white matter boundaries (white matter surface) to gray matter and cerebrospinal fluid boundaries (pial surface) on the cortex in each hemisphere. All participants’ images were visually inspected for excessive motion, signal drop-out, and/or other artifacts. 2. DTI data: Diffusion-weighted images were preprocessed in FSL (Andersson et al., 2003; Andersson and Sotiropoulos, 2016; Smith et al., 2004), MRtrix3 (Tournier et al., 2019), and TRActs Constrained by UnderLying Anatomy (TRACULA) (Maffei et al., 2021; Yendiki, 2011). For each participant, the DWI images were skull stripped using FSL bet, corrected for distortion, head motion, and eddy current using FSL topup and eddy tools. Additionally, they were denoised and bias field corrected using dwidenoise and dwibiascorrect in MRtrix3. The final corrected DWI was used as an input to TRACULA, a global probabilistic automatic tractography algorithm in FreeSurfer 7.2.0 (Maffei et al., 2021; Yendiki, 2011). TRACULA is capable of reconstructing 42 major white matter tracts, including the fornix which required the segmentation of the thalamic subnuclei (Iglesias et al., 2018). A detailed workflow of the TRACULA algorithm has been described elsewhere (Yendiki, 2011; Yendiki et al., 2016). The processing steps included: (1) cortical and subcortical segmentation of T1-weighted image using FreeSurfer as previously described above; (2) within-subject registration of DTI to T1-weighted (Greve and Fischl, 2009); (3) between-subject registration in Advanced Normalization tool (ANTs) (Avants et al., 2008) to map each participant onto an Fractional Anisotropy (FA) template constructed from a training dataset in order to ensure that the relative position of the anatomical structures was the same for all participants and to map median streamline from the training data to the participant during initialization (Maffei et al., 2021); and (4) the applications of tensor fitting for the extraction of tensor-based measures using DTIFIT and ball and stick model using BEDPOSTX (Behrens et al., 2003, 2007). Finally, the probabilistic reconstruction of 42 major white matter tracts. Importantly, step 3 was only used to initialize the reconstruction of the tracts, which was then refined by fitting them to the anatomy of the individual participant.

In this study, we were interested in the following region of interest (ROI) tracts because of their involvement in visuomotor processing and their susceptibility to brain injury (Hurtubise et al., 2020; Mustafi et al., 2022): the CC body (parietal and premotor), CC splenium, middle cerebellar peduncle (MCP), and bilateral CST, ILF, and SLF 1 and 2 (Figure 2b–f). We visually inspected the above tracts for all participants. Tract reconstructions were considered successful if they transverse the relevant WM regions and reached the cortical regions that were used as inclusion ROIs in the protocols defined to manually label the training set (Maffei et al., 2021). Regarding the tensor-based measures, we extracted the FA and Mean Diffusivity (MD) values averaged over each entire tract in addition to the FA and MD averaged at consecutive cross-sections of each tract. The latter resulted in an along-tract profile for each tensor measure. The along-tract profiles for tensor measures were obtained using a pointwise assessment of streamline tractography attributes (PASTA), which is a type of analysis where an along-tract profile of a microstructural measure (e.g., FA) is generated by averaging the values of the measure at different cross-sections of a tract (Jones et al., 2005). For each of the ROI tract, a 1D along-tract profile of FA and MD were generated by projecting the value of each measure from every point on every automatically reconstructed streamline to its nearest point on a reference streamline (Maffei et al., 2021). The reference streamline was the mean of the training streamlines for each tract in template space, ensuring that all participants’ data were sampled at the same number of cross-sections along a given bundle. The length of each 1D profile was the length of the reference streamline, i.e., the average length of the manually annotated streamline in template space. 3. rsfMRI for Functional Connectivity: Multi-echo rsfMRI pre-processing was done using the Multi-Echo Independent Components Analysis (ME- ICA) pipeline in the Analysis of Functional NeuroImages (AFNI) software (Cox, 1996; Kundu et al., 2017). ME- ICA is a method for fMRI analysis and denoising based on the T2* decay of BOLD signals, as measured using multi-echo fMRI. ME-ICA decomposes multi-echo fMRI datasets into independent components (ICs) using FastICA, then categorizes ICs as BOLD or noise using their BOLD and non-BOLD weightings (measured as Kappa and Rho values, respectively) (Kundu et al., 2017). Prior to denoising with ME-ICA, the functional data preprocessing steps included discarding the first 5 volumes of each rsfMRI time-series. Images were then skull- stripped and image intensity is normalized (3dSkullStrip). The functional images were de-obliqued (3dWarp). Large signal transients were removed via interpolation (“despiking,” 3dDespike) and slice time correction was applied (3dTshift). Motion correction parameters were calculated using the middle echo (TE2 = 30.15 ms, 3dvolreg). The skull-stripped anatomical and functional images were co-registered using the first volume of the middle echo images (3dAllineate). Optimal combination of the 3 echo times was performed prior to initializing ME-ICA denoising. In ME-ICA, BOLD signal was identified as independent components having linearly TE-dependent percentage signal changes. Non-BOLD noise components were removed from the time-series by ME-ICA using linear regression. The output of this process included a functional time-series that was reconstructed to include only the BOLD signal components of the data. All participants’ preprocessed functional images were visually inspected for excessive motion, co-registration errors, and to verify that the functional data were not excessively noisy (a minimum of 10 independent components classified as BOLD signal by ME-ICA).

#### 2.4.2 Functional parcellation

After ME-ICA, the final preprocessed and denoised time-series (“participantID_medn.nii.gz”) was used as input data for Group prior individual parcellation (GPIP) analysis. GPIP is an individualized functional parcellation approach that was used here to identify participant-specific functional resting network nodes in the preprocessed data (Chong et al., 2017). Network parcellations refers to the identification of cortical areas that exhibit functionally similar properties (Kim et al., 2010; Raichle et al., 2001; Smith et al., 2013; Sporns et al., 2005). The most common approach to parcellation relies on a mean functional resting state network parcellation common to a group of participants (i.e., the group average network), which is then projected back onto individual participant data (Thomas Yeo et al., 2011; Wig et al., 2014). These population-average networks have provided important information on the large-scale functional organization of the brain (Buckner et al., 2013; Wig, 2017). However, population-average networks may obscure participant-specific network organization and thus lead to inaccuracies at the level of each participant (Chong et al., 2017). Thus, there is a growing focus on person-specific parcellation to define functional parcels independently for each participant. Group prior individual parcellation (GPIP) was implemented to automatically perform parcellation of resting functional data into functional networks at the participant level (Chong et al., 2017). GPIP is a novel cortical parcellation method that initializes parcellation using an atlas template. This initial parcellation is then refined for each participant using the participant’s functional data to allow individual variability across participants in the boundaries of these parcels (Chong et al., 2017). The use of a template atlas for parcellation initialization results in all participants having corresponding functional regions (aiding group analysis), while functional parcel boundaries can vary from participant to participant. GPIP iterates between two steps to continuously update parcel labels until convergence: (1) each participant’s parcel boundaries (first obtained from the initialization to the Schaefer atlas) are refined relative to their resting functional data, and (2) the concentration (inverse covariance/partial correlation) matrices from all individuals are then jointly estimated using a group sparsity constraint (Chong et al., 2017). Specifically, for the results presented in the current study, the preprocessed and denoised resting state functional data were first initialized with the 200-parcel Schaefer atlas (Schaefer et al., 2018), corresponding to the 7 functional networks atlas (Thomas Yeo et al., 2011).

Prior to GPIP initialization, the denoised functional data were registered to the corresponding T1- weighted FreeSurfer anatomical images for each participant, converted from volumetric to surface space, and resampled to the FreeSurfer cortical surface template (fsaverage5). Spatial smoothing of 6 mm was applied to the anatomically- aligned data in surface space. Visual inspection was used to verify proper co-registration of functional data with the T1-weighted anatomical images. Values from vertices located in the medial wall were resampled into the surface data as they are removed by FreeSurfer but are needed for running GPIP. The functional time-series data were normalized by scaling to a mean value of 0 and a standard deviation of 1. The normalized functional time-series data were then used in subsequent steps for GPIP analysis. GPIP performed its two-step iterative process 20 times for each subject resulting in increasingly refined functional network parcellations with optimal segmentation with respect to the cortical surface. Each participant’s final parcellation was plotted and inspected to verify the quality of the parcellation. To further assess the quality of the parcellations, homogeneity was calculated as the mean temporal correlation coefficient between all pairs of vertices within each GPIP parcel, where a large value suggests that the vertices included in a particular parcel have similar time-series (i.e., are homogeneous) and therefore correctly assigned to that parcel. The homogeneity value was calculated for the whole brain as a mean value across all parcels for each GPIP iteration to verify that these values increased with each iteration before plateauing prior to the final iterations, suggesting stable and accurate parcellations. Further, cross-correlation matrices including all GPIP parcels were plotted and visually inspected to verify reasonable patterns of whole-brain connectivity in each participant (see section “Resting state functional connectivity matrix” below for specific details on matrix construction).

#### 2.4.3 Resting state functional connectivity matrix

**Figure 3:**
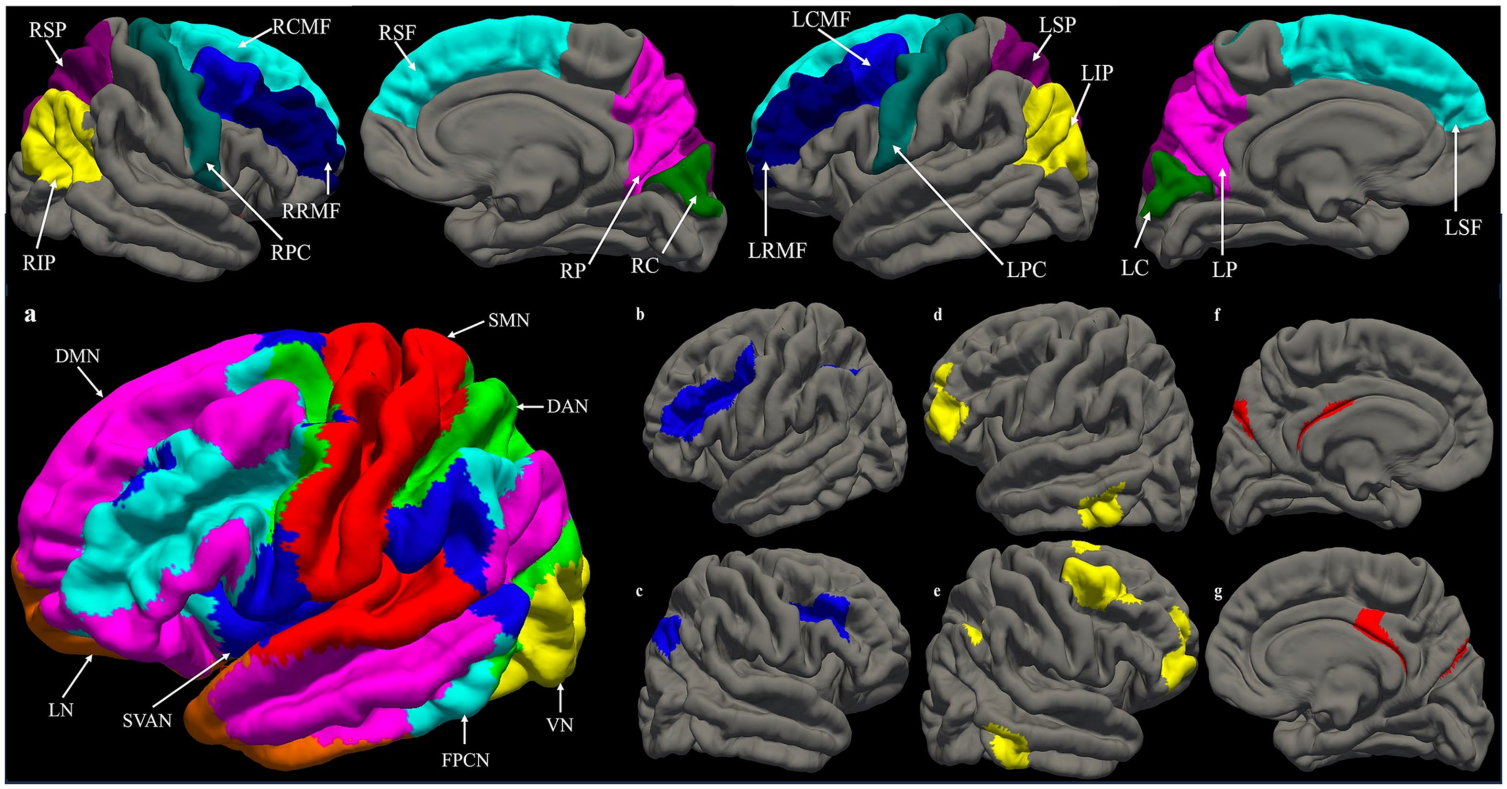
Top panel: Cortical regions of interest. RSP, right superior parietal; RIP, right inferior parietal; RPC, right precentral; RRMF, right rostral middle frontal; RCMF, right caudal middle frontal; RSF, right superior frontal; RP, right precuneus; RC, right cuneus. LSP, right superior parietal; RIP, right inferior parietal; RPC, right precentral; RRMF, right rostral middle frontal; RCMF, right caudal middle frontal; RSF, right superior frontal; RP, right precuneus; RC, right cuneus. LSP, left superior parietal; LIP, left inferior parietal; LPC, left precentral; LRMF, left rostral middle frontal; LCMF, left caudal middle frontal; LSF, left superior frontal; LP, left precuneus; LC, left cuneus. Bottom panel: **(a)** Resting functional connectivity networks. DAN, dorsal attention network; DMN, default mode network; FPCN, frontoparietal network; LN, limbic network; SMN, sensorimotor control network; SVAN, salience ventral attention network; VN, visual network. The limbic network was excluded from analyses. **(b–g)** The 3 subnetworks of the frontoparietal control network (FPCN), **(b)** left FPCNa; **(c)** right FPCNa; **(d)** left FPCNb; **(e)** right FPCNb; **(f)** left FPCNc; **(g)** right FPCNc.

A functional connectivity matrix was created for each participant based on their individualized parcellation. The mean BOLD signal time-series data was extracted from each parcel and pairwise correlations were computed between each parcel pair. The correlation coefficients were then converted to z-scores using Fisher’s r-to-z transformation to normalize the distribution of correlation values, resulting in a 200 × 200 functional connectivity matrix for each participant. Each participant’s mean Fisher z-transformed RSFC values were extracted for 6 resting state networks of interest as a measure of overall within-network functional connectivity. The networks of interest were the visual network (VN), sensorimotor control network (SMN), dorsal attention network (DAN), SVAN, FPCN, and DMN (Figure 3a–g). Furthermore, the FPCN was separated into 3 subnetworks based on the FPCN from the Schaefer et al. (2018) 17-networks parcellation. We chose to divide the FPCN in subnetworks because a growing body of research has identified two functional cores of the FPCN: FPCNa which has stronger connectivity with the DMN and FPCNb which showed stronger connectivity with the DAN (Beaty et al., 2021; Dixon et al., 2018; Murphy et al., 2020; Yin et al., 2022). DAN plays a key role in visuospatial perceptual attention and visually-guided reaching actions (Ptak, 2012; Ptak et al., 2017; Thomas Yeo et al., 2011) and has a close relationship with SMN, while DMN is independent of sensory input and involved in introspective processes (Konishi et al., 2015). The division was done by matching the FPCN MNI centroid coordinates from the 7 networks atlas to the corresponding FPCN MNI centroid coordinates from the 17 networks atlas (Figure 3b). Although, the third subnetwork (FPCNc: the posterior cingulate and precuneus) did not contain any frontal components, we included it because there is evidence showing altered functional connectivity post-concussion (Leech and Sharp, 2014).

### 2.5 Statistical analyses

With exception of cortical thickness and along-tract DTI analyses, all statistical analyses were conducted using R (v 3.4.1), and scatter and box plots were generated using the ggplot2 package. Data were checked for normality by visual inspection of the distributions, Shapiro–Wilk’s test and Q-Q plots of the residuals. One participant was excluded from functional connectivity and cortical thickness analyses because no MRI was acquired (*n* = 21), while 2 participants were removed from DTI analyses based on the aforementioned reason and analysis pipeline failure (*n* = 20). Descriptive statistics were used to summarize participants’ characteristics. A paired *t*-test was used to detect any significant differences in timing and trajectory composite scores, RT, PLf, and %DR between standard and PC + FR conditions. Model-based analyses involving RPQ and DHI were adjusted for age, sex, experience with competitive and/or recreational sports, and days since last concussion while we adjusted for age, sex, experience with competitive and/or recreational sports, experience with video games, and days since last concussion for all model- based analyses involving visuomotor performance. The adjusted variables were selected based on previous findings that revealed significant predictors of visuomotor performance and PPCS severity (Hurtubise et al., 2016; Sergio et al., 2020; Smeha et al., 2022). Also, model-based measures were reported as effect estimates and 95% confidence intervals (CI). Note that with the RPQ scale, 1 indicates that the symptom was no longer present, while 0 indicates that the symptom was never present. Hence this measure may not be strictly ordinal. We felt symptom history was an important metric with potential neurophysiological significance, however, and kept the measure as collected for our analyses.

#### 2.5.1 Relationship between cortical thickness, RPQ, DHI, and Visuomotor performance

Whole brain and ROI vertex-wise general linear model (GLM) analyses were conducted using FreeSurfer’s built-in mri_glmfit tool to investigate the associations between cortical thickness, PPCS severity (RPQ-3 and 13), dizziness-related symptoms (DHI: emotional, physical, functional, and total), and visuomotor performance measures (standard and PC + FR conditions). The ROIs were obtained from the Desikan-Killany atlas (Desikan et al., 2006) based on their involvement in the FPCN for visually-guided reaching (Hurtubise et al., 2022): bilateral superior parietal and inferior parietal cortices, precuneus, precentral, superior frontal, rostral middle frontal, caudal middle frontal, and cuneus (Figure 3, top panel). All participants’ images were resampled to a common space (fsaverage) and smoothed with a 10-mm full-width half-maximum (FWHM) kernel before GLM analyses. A z-distribution Monte Carlo simulation with 5,000 iterations using a cluster-forming threshold of 2 (*p* = 0.01) and cluster-wise *p* < 0.05 were used for multiple comparisons correction (mri_glmfit-sim). Bonferroni correction was applied across both hemispheres.

#### 2.5.2 Relationship between RSFC, RPQ, DHI, and visuomotor performance

Multiple linear regression models were set up with RSFC as the independent variable and RPQ, DHI, or visuomotor performance measures as dependent variable. The independent variable was the mean within- network functional connectivity of the VN, SMN, DAN, SVAN, FPCN, and DMN as well as the FPCN subnetworks (FPCNa, FPCNb, and FPCNc).

#### 2.5.3 Relationship between DTI measures, RPQ, DHI, and visuomotor performance

We tested the along-tract FA or MD values for associations with PPCS severity, dizziness-related symptoms, and visuomotor performance measures. We fitted a GLM at each point along each tract using FreeSurfer’s mri_glmfit tool that was adapted for 1D data. A permutation-based 5,000 simulations was used for multiple comparisons correction with both the cluster-forming threshold and cluster-wise set at *p* = 0.05. Bonferroni correction was applied across both hemispheres. Furthermore, multivariate multiple linear regression analyses were set up with FA or MD values averaged over an entire tract as the independent variable and RPQ, DHI, or visuomotor performance measures as the dependent variable.

All *p*-values obtained from multiple linear regression analyses were adjusted for multiple comparisons using the Holm correction method and were considered statistically significant at *p* < 0.05 at the level of each regression analysis.

## 3 Results

### 3.1 Participant characteristics

Participant’s demographic, PPCS, dizziness, and visuomotor characteristics are displayed in Table 1. There was a higher percentage of females (77.27%), and the mean age was 47.23 years old. The median age was 48.5, with a range of 29–63 years old. Of the 22 participants, 5 reported having 1 concussion (22.73%), 3 reported having 2 concussions (13.64%), 8 reported having 3 concussions (36.36%), and 6 reported having 4 or more concussions (27.27%). All participants reported experiencing poor concentration (Table 2). Dizziness, forgetfulness, taking longer to think were the second most reported symptoms (95.45% respectively), while double vision was the least reported symptom (50%) (Table 2). During data collection, 8 participants reported full-time employment, 8 reported part-time employment, 1 reported mixed employment (full-time and part-time), 3 reported no employment before and after experiencing concussion, and 2 reported full-time employment prior to concussion and unemployed after concussion. Regarding the mechanisms of concussion, motor vehicle accident had the highest frequency (30.16%), followed by sports (25.39%), and fall and projectile objects (19.05 and 19.05%, respectively). Other mechanisms included fights, bicycle and elevator accidents, and mixed mechanisms (sports and projectile object) which accounted for 6.36% combined.

**Table 1:** Study participants characteristics (*n* = 22).

Demographics	
Age	47.23 ± 9.26
Sex, *n* (%) male	5 (22.73)
Education (yrs)	16.77 ± 1.99
Days since last concussion	1795.13 ± 963.11
Average number of concussion	3.05 ± 1.76
Loss of consciousness, yes (%)*	15.87
Dazed and confused, yes (%)*	92.06
No memory for events immediately after the injury, yes (%)*	28.33
PPCS severity	
RPQ-3	7.18 ± 2.22
RPQ-13	32.68 ± 10.26
Dizziness severity	
DHI-physical	11.27 ± 4.43
DHI-functional	20.09 ± 10.26
DHI-emotional	13.36 ± 7.23
DHI-total	44.73 ± 20.45
Depression severity	
PHQ-9	12.05 ± 6.59
Visuomotor metrics	
Standard timing composition score	1.46e-15 ± 2.61
Standard trajectory composite score	4.04e−16 ± 1.76
Standard reaction time (ms)	777.50 ± 657.64
Standard full path length (mm)	54.25 ± 1.88
PC + FR timing composition score	−5.75e−16 ± 1.69
PC + FR trajectory composition score	1.71e−16 ± 1.82
PC + FR DR	31.60 ± 22.38
PC + FR reaction time (ms)	476.14 ± 302.88
PC + FR full path length (mm)	74.43 ± 31.02

All data are presented in mean (SD) unless otherwise indicated. DHI, Dizziness Handicap Inventory; PHQ, Patient Health Questionnaire; PPCS, Persistent Post-Concussion Syndrome; PC + FR, Plane Change + Feedback Reversal; RPQ, Rivermead Post-Concussion Symptoms Questionnaire. *Across all number of concussions.

**Table 2:** Frequency of endorsed persistent symptoms after concussion based on rating of 2 and greater* on the Rivermead Post-concussion Symptom Questionnaire (RPQ).

Symptom	*n* (%)	Mean ± SD
Headaches	19 (86.36)	3.26 ± 0.73
Dizziness	21 (95.45)	2.67 ± 0.86
Nausea and/or vomiting	14 (63.64)	2.36 ± 0.63
Noise sensitivity, easily upset by loud noise	20 (90.91)	3.45 ± 0.69
Sleep disturbance	18 (81.82)	3.17 ± 0.86
Fatigue, tiring more easily	18 (81.82)	3.56 ± 0.70
Being irritable, easily angered	17 (77.27)	3.18 ± 0.88
Feeling depressed or tearful	12 (54.55)	2.75 ± 0.75
Feeling frustrated or impatient	18 (81.82)	2.73 ± 0.83
Forgetfulness, poor memory	21 (95.45)	3.33 ± 0.73
Poor concentration	22 (100.00)	3.09 ± 0.75
Taking longer to think	21 (95.45)	3.14 ± 0.79
Blurred vision	14 (63.64)	2.93 ± 0.83
Light sensitivity, easily upset by bright light	18 (81.82)	3.00 ± 0.84
Double vision	11 (50.00)	2.55 ± 0.69
Restlessness	12 (54.55)	2.67 ± 0.49

*Rating of 2 = a mild problem.

### 3.2 Cognitive-visuomotor behavior

**Figure 4:**
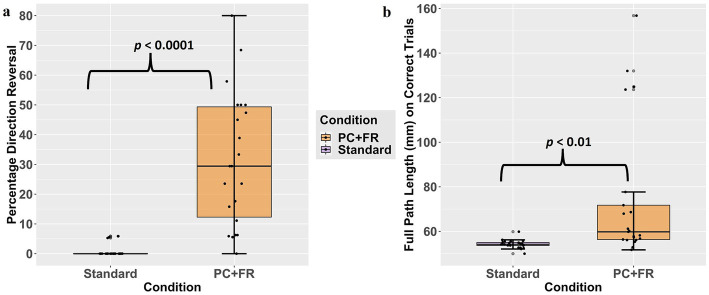
Results from paired *t*-test between standard and PC + FR conditions on **(a)** Percentage direction reversal and **(b)** Full path length. PC + FR, plane change and feedback reversal. PC + FR, plane-change + feedback reversal.

There were no significant differences between and PC + FR conditions on timing and trajectory composite scores and reaction time (RT) (timing: *t* = 0.184, *p* = 0.856; trajectory: *t* = 0.156, *p* = 0.878; RT: *t* = −1.64, *p* = 0.117). However, the PC + FR condition showed significantly more direction reversal (DR) and longer full path length (PLf) compared to the standard condition as expected (DR: *t* = 6.40, *p* < 0.0001; PLf: *t* = 3.10, *p* < 0.01) (Figure 4).

### 3.3 Relationship between cortical thickness, symptoms, and visuomotor performance

**Figure 5:**
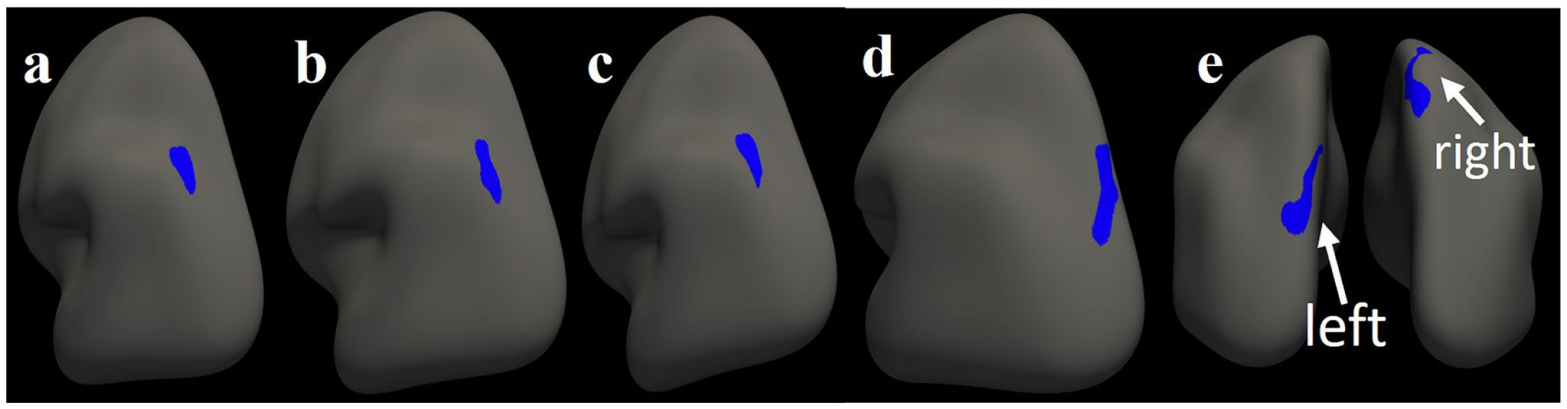
Significant clusters from region of interest analyses overlaid on a left inflated surface. The blue color depicts a negative association between left inferior parietal gyrus thickness with **(a)** DHI functional domain; **(b)** DHI emotional domain; **(c)** DHI total score. DHI, Dizziness Handicap Inventory. Significant clusters from region of interest analyses overlaid on a left and right inflated surfaces. The blue color depicts **(d)** negative association between cortical thickness cluster in left superior parietal gyrus and timing composite score in PC + FR condition; **(e)** negative association between cortical thickness clusters in bilateral superior parietal gyri thickness and reaction time in PC + FR condition. DHI, Dizziness Handicap Inventory; PC + FR, plane change and feedback reversal.

Cortical thickness ROI analysis results are summarized in Table 3 and Figure 5a. Higher scores on the functional and emotional DHI domains and total DHI were associated with decreased cortical thickness in the left inferior parietal gyrus, respectively, such that as thickness decreased, the severity of dizziness- reported symptoms increased. No associations were observed on whole-brain analysis. Lastly, we observed no relationships between RPQ-3 and 13 and cortical thickness.

**Table 3:** Brain regions showing a significant relationship between cortical thickness, dizziness-related symptoms, and visuomotor performance.

Measures	Regions	MNI coordinates *x, y, z*	Cluster size (mm^2^)	Cluster-wise *p-*value	Confidential interval
DHI					
Functional	Left inferior parietal gyrus	−30.2, −66.5, 41.0	329.31	0.048	0.043–0.054
Emotional	Left inferior parietal gyrus	−32.7, −77.5, 39.9	354.35	0.036	0.031–0.041
Total	Left inferior parietal gyrus	−31.0, −66.4, 41.3	337.21	0.045	0.039–0.050
PC+FR condition					
Timing score	Left superior parietal gyrus	−19.6, −79.5, 42.2	347.40	0.035	0.029–0.039
Reaction time	Left superior parietal gyrus	−16.6, −86.4, 31.8	400.71	0.011	0.008–0.014
Reaction time	Right superior parietal gyrus	9.3, −64.2, 59.6	345.39	0.033	0.028–0.037

MNI coordinates reflect location of peak voxel within cluster. DHI, dizziness handicap inventory; MNI, Montreal neurological institute; PC+ FR, plane change and feedback reversal.

In the PC + FR condition, ROI analysis showed negative associations between timing score and left superior parietal gyrus thickness (Table 3; Figure 5b). In all regions, a decrease in thickness was associated with an increase in visuomotor score, implying worse performance. No associations were observed on whole-brain analysis. Further, we found no associations between standard condition visuomotor performance and cortical thickness.

### 3.4 Resting state functional connectivity, symptoms, and visuomotor performance

**Figure 6:**
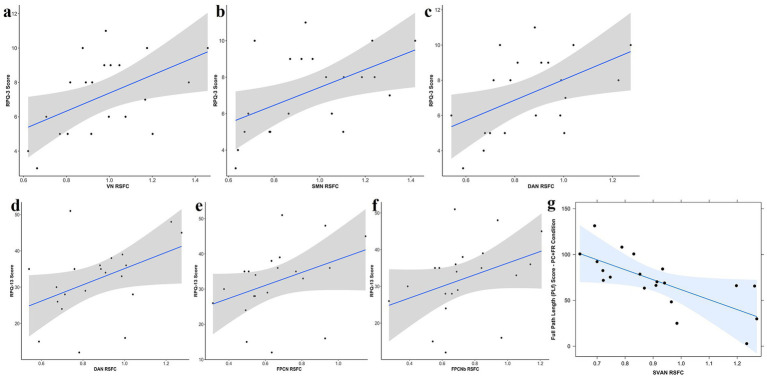
Increased early persistent PCS cluster was associated with higher RSFC in **(a)** VN; **(b)** SMN; **(c)** DAN. DAN, dorsal attention network; SMN, sensorimotor control network; RSFC, resting state functional connectivity; RPQ-3, Rivermead post-concussion symptoms questionnaire cluster 1; VN, visual network. **(d–f)** Relationship between mean intra-networks RSFC and RPQ-13 score. Increased in later psychological and cognitive persistent PCS cluster was associated with higher RSFC in **(a)** DAN; **(b)** FPCN; **(c)** FPCNb. DAN, dorsal attention network; FPCN, frontoparietal control network; FPCNb, frontoparietal control network-subnetwork b; RSFC, resting state functional connectivity; RPQ-13, Rivermead post-concussion symptoms questionnaire cluster 2. **(g)** Relationship between mean intra-SVAN RSFC and full path length in PC + FR condition. PC + FR, plane change and feedback reversal; RSFC, resting state functional connectivity; SVAN, salient ventral attention network.

In RPQ-3, higher mean RSFC within the VN, SMN and DAN were associated with increased early physical symptoms cluster of PPCS (Table 4; Figures 6a–c). In RPQ-13, higher mean RSFC within the DAN and FPCN were associated with increased later psychological and cognitive symptoms cluster of PPCS (Table 4; Figures 6d–f). Lastly, we did not observe any associations between mean RSFC within-networks and DHI. Behaviorally, we observed that a shorter PLf in the PC + FR condition, indicating a better performance, was associated with higher mean RSFC within SVAN (Table 4; Figure 6g).

**Table 4:** Linear regression models used to assess the relationship between resting state functional connectivity (RSFC), persistent PCS, and visuomotor performance.

Exposure variables	Beta coefficient	*p*-value
Outcome variable: RPQ3		
*R*^2^ = 0.31		
Sex (female compared to male)	1.23	0.26
Visual network	5.99	0.12
*R*^2^ = 0.37		
Sex (female compared to male)	1.88	0.10
SomMot	6.487	0.01
*R*^2^ = 0.28		
Sex (female compared to male)	0.94	0.39
DorsAttn	6.29	0.02
Outcome variable RPQ-13		
*R*^2^ = 0.25		
Sex (female compared to male)	8.74	0.12
FPCN_B	23.42	0.03

### 3.5 White matter integrity, symptoms, and visuomotor performance

**Figure 7:**
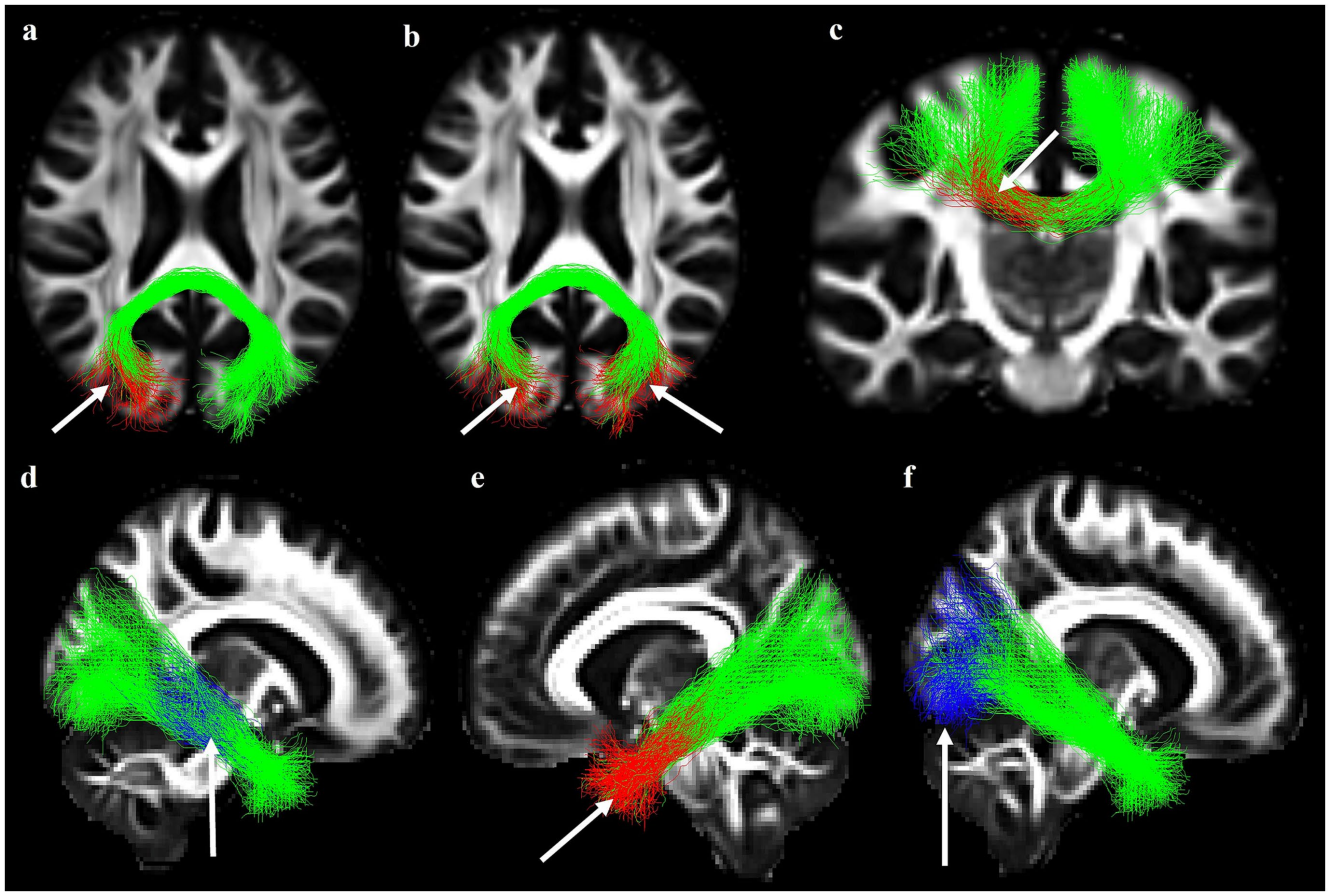
Significant positive associations from along-tract MD analysis with RPQ and DHI scores. White arrows indicate affected areas along each white matter tract: **(a)** RPQ-3 and CC splenium; **(b)** DHI functional domain and CC splenium; **(c)** DHI functional and CC body-premotor. Significant associations from along-tract FA and MD analyses with visuomotor performance. White arrows indicate affected areas along each white matter tract: **(d)** negative association between standard condition and right ILF FA; **(e)** positive association between standard condition and left ILF MD; **(f)** negative association between PC + FR condition and right ILF FA. CC, corpus callosum; DHI, dizziness handicap inventory; RPQ, Rivermead post-concussion symptoms questionnaire. D. Higher MD in the entire right SLF-2 was associated with increased early persistent PCS cluster. MD, mean diffusivity; SLF-2, superior longitudinal fasciculus 2; RPQ-3, Rivermead post-concussion symptoms questionnaire cluster 1. FA, fractional anisotropy; ILF, inferior longitudinal fasciculus; MD, mean diffusivity; PC + FR, plane change and feedback reversal.

**Figure 8:**
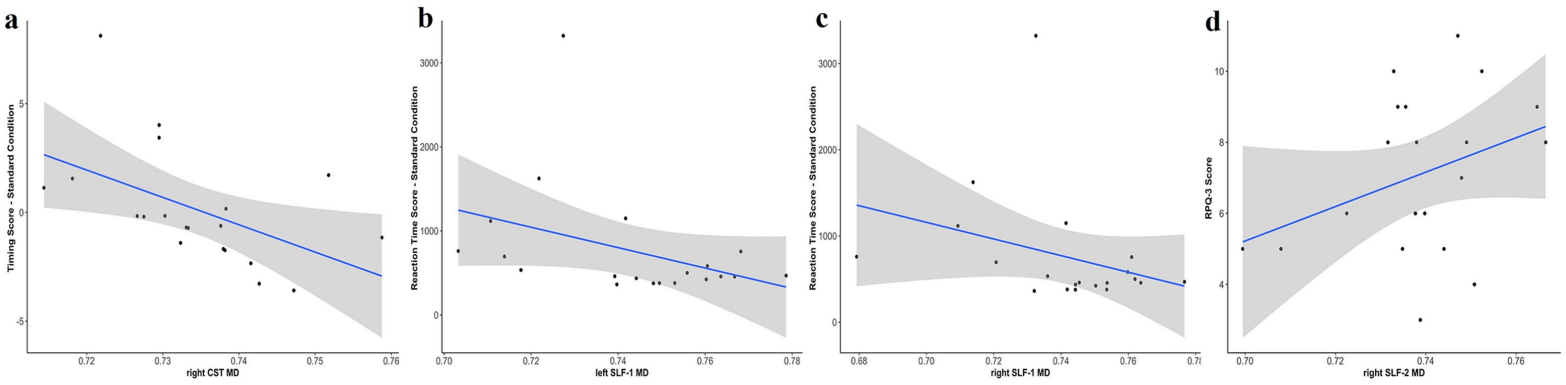
Relationship between MD in the entire-tract and standard condition. **(a)** Right CST and timing score; **(b)** left SLF-1 and reaction time score; **(c)** right SLF-1 and reaction time score; **(d)** higher MD in the entire right SLF-2 was associated with increased early persistent PCS cluster CST, corticospinal tract; MD, mean diffusivity; SLF-1, superior longitudinal fasciculus 1, MD, mean diffusivity; SLF-2, superior longitudinal fasciculus 2; RPQ-3, Rivermead Post-Concussion Symptoms Questionnaire cluster 1.

An examination of Along-tract and Entire-tract FA and MD, and the relationship to symptom scores for the RPQ and DHI, revealed a number of significant relationships. First, we found a positive association between RPQ-3 and CC splenium MD (Table 5; Figure 7a) and positive associations between DHI functional domain, CC splenium MD, and CC body-premotor MD (Table 5; Figures 7b,c). Next, we examined relationships between Along-tract and Entire-tract FA and MD and Visuomotor performance. In the standard condition, the along-tract analysis revealed that the timing score was negatively associated with right ILF FA (Table 5; Figure 7d) and positively associated with left ILF MD (Table 5; Figure 7e). Furthermore, the following associations were obtained from the entire-tract analysis: timing score vs. right CST MD (Table 6; Figure 7f) and RT vs. bilateral SLF-1 MD (Table 5; Figures 8a,b). Finally, in the PC + FR condition, we found a negative association between the timing score and right ILF FA on the along-tract analysis (Table 5; Figure 8c).

**Table 5:** White matter tracts showing a significant relationship between along-tract measures, persistent PCS, dizziness-related symptoms, and visuomotor performance.

Measures	Regions	Voxel coordinates *x, y, z*	Cluster size (mm^3^)	Cluster-wise *p-*value	Confidential interval (CI)
RPQ					
RPQ-3	CC splenium MD	72.00, 0.00, 0.00	40.5	0.019	0.017–0.022
DHI					
Functional	CC splenium MD	72.00, 0.00, 0.00	33.8	0.035	0.032–0.039
	CC splenium MD	6.00, 0.00, 0.00	33.8	0.035	0.032–0.039
	CC body-premotor MD	46.00, 0.00, 0.00	47.2	0.046	0.043–0.050
Standard condition					
Timing score	Right ILF FA	57.00, 0.00, 0.00	54.0	0.018	0.014–0.021
	left ILF MD	74.00, 0.00, 0.00	74.2	0.009	0.008–0.012
PC+FR condition					
Timing score	Right ILF FA	6.00, 0.00, 0.00	50.6	0.023	0.019–0.027

Voxel coordinates reflect location of peak voxel within cluster. DHI, dizziness handicap inventory; PC+ FR, plane change and feedback reversal; RPQ, rivermead post-concussion symptoms questionnaire; CC, corpus callosum; MD, mean diffusivity; ILF, inferior longitudinal fasciculus; FA, fractional anisotropy.

**Table 6:** Linear regression models used to assess the relationship between entire white matter tracts measures, persistent PCS, and visuomotor performance.

Outcome	Independent variables	Unstandardized B	S. E.	Unadjusted *p*-value	Adjusted *p*-value
RPQ-3					
	Right SLF-2 MD	106.750	28.801	**0.002**	**0.009**
	Sex: female	−0.831	1.046	0.441	0.959
	Age	0.213	0.055	0.002	0.008
	Days since last concussion	−0.001	0.001	0.319	0.959
	Sport experience	0.099	0.389	0.804	0.959
Standard condition					
Timing score	Right CST MD	−98.568	33.308	**0.012**	0.059
	Sex: female	−1.817	0.892	0.064	0.193
	Age	0.241	0.047	*p* < 0.001	0.002
	Days since last concussion	−0.001	0.0004	0.064	0.193
	Sport experience	−0.200	0.386	0.613	0.613
	Video game experience	0.642	0.225	0.015	0.059
RT	Left SLF-1 MD	−14389.378	6488.375	**0.047**	0.154
	Sex: female	−743.888	304.884	0.031	0.154
	Age	55.072	16.284	0.005	0.033
	Days since last concussion	−0.227	0.137	0.125	0.249
	Sport experience	−20.555	130.009	0.877	0.877
	Video game experience	184.348	78.109	0.036	0.154
	Intercept	10961.562	5159.528	0.055	–
	Right SLF-1 MD	−16148.645	6661.704	**0.032**	0.114
	Sex: female	−818.289	293.573	0.016	0.082
	Age	55.101	15.794	0.004	0.027
	Days since last concussion	−0.169	0.129	0.214	0.428
	Sport experience	−59.668	124.525	0.640	0.640
	Video game experience	189.836	76.276	0.028	0.114
PC+FR condition					
Trajectory score	Right CST FA	−52.428	15.092	0**.005**	**0.028**
	Sex: female	0.233	0.708	0.748	1.000
	Age	−0.008	0.037	0.834	1.000
	Days since last concussion	0.0001	0.0003	0.705	1.000
	Sport experience	0.341	0.301	0.279	
	Video game experience	−0.469	0.181	0.023	0.116
PLf	Right CST FA	−839.299	342.312	**0.031**	0.183
	Sex: female	−37.211	16.056	0.039	0.195
	Age	1.877	0.847	0.047	0.195
	Days since last concussion	−0.002	0.007	0.780	1.000
	Sport experience	0.590	6.823	0.933	1.000
	Video game experience	1.341	4.094	0.749	1.000
	left ILF MD	−735.327	296.583	**0.029**	0.174
	Sex: female	−27.262	16.419	0.123	0.614
	Age	1.389	0.852	0.129	0.614
	Days since last concussion	−0.004	0.007	0.557	1.000
	Sport experience	6.617	7.048	0.366	1.000
	Video game experience	0.739	4.054	0.858	1.000
	left SLF-1 MD	−802.793	353.812	**0.043**	0.255
	Sex: female	−31.588	16.593	0.081	0.406
	Age	1.282	0.888	0.174	0.697
	Days since last concussion	−0.007	0.007	0.381	1.000
	Sport experience	4.728	7.089	0.517	1.000
	Video game experience	2.093	4.259	0.632	1.000
	left SLF-2 MD	−1105.439	416.987	**0.021**	0.127
	Sex: female	−28.746	15.888	0.096	0.478
	Age	1.444	0.829	0.107	0.478
	Days since last concussion	−0.007	0.007	0.331	0.873
	Sport experience	7.710	6.982	0.291	0.873
	Video game experience	4.023	4.229	0.360	0.873
	Intercept	1175.812	374.669	0.009	-
	Right SLF-2 MD	−1565.452	505.139	**0.009**	0.055
	Sex: Female	−32.663	14.709	0.046	0.232
	Age	1.336	0.782	0.113	0.453
	Days since last concussion	0.004	0.007	0.573	0.789
	Sport experience	5.587	6.329	0.395	0.789
	Video game experience	6.186	4.207	0.167	0.502
Timing score	Right CST FA	44.128	19.952	**0.047**	0.283
	Sex: female	0.486	0.936	0.613	1.000
	Age	0.095	0.049	0.079	0.397
	Days since last concussion	−0.001	0.0004	0.100	0.401
	Sport experience	0.074	0.398	0.856	1.000
	Video game experience	0.084	0.239	0.732	1.000
RT	Right CST FA	10174.886	2814.487	**0.004**	**0.021**
	Sex: female	348.622	132.016	0.022	0.108
	Age	−6.429	6.965	0.374	0.969
	Days since last concussion	−0.026	0.058	0.658	0.969
	Sport experience	57.832	56.102	0.323	0.969
	Video game experience	−64.088	33.666	0.081	0.325
	Right SLF-2 MD	13026.401	5392.922	**0.033**	0.196
	Sex: female	307.959	157.041	0.074	0.294
	Age	−1.364	8.347	0.873	1.000
	Days since last concussion	−0.074	0.072	0.326	0.978
	Sport experience	11.523	67.579	0.867	1.000
	Video game experience	−99.348	44.918	0.047	0.236
% DR	Right CST MD	831.019	349.011	**0.035**	0.139
	Sex: female	−17.943	9.348	0.079	0.237
	Age	2.712	0.497	*p* < 0.001	*p* < 0.001
	Days since last concussion	−0.013	0.004	0.008	0.038
	Sport experience	6.320	4.042	0.144	0.289
	Video game experience	1.219	2.361	0.615	0.615

Unadjusted and adjusted *p*-values reported. Values were adjusted for multiple comparisons using Holm correction method and considered significant at *p* < 0.05. PC+FR, plane change and feedback reversal; PLf, full path length; RPQ, rivermead post-concussion symptoms questionnaire; S. E, standard errors; RT, reaction time; DR, direction reversal; MD, mean diffusivity; ILF, inferior longitudinal fasciculus; FA, fractional anisotropy; CST, corticospinal tract; SLF-1 and-2, superior longitudinal fasciculus 1 and 2. Bold values highlight significant relationships at *α* = 0.05.

**Figure 9:**
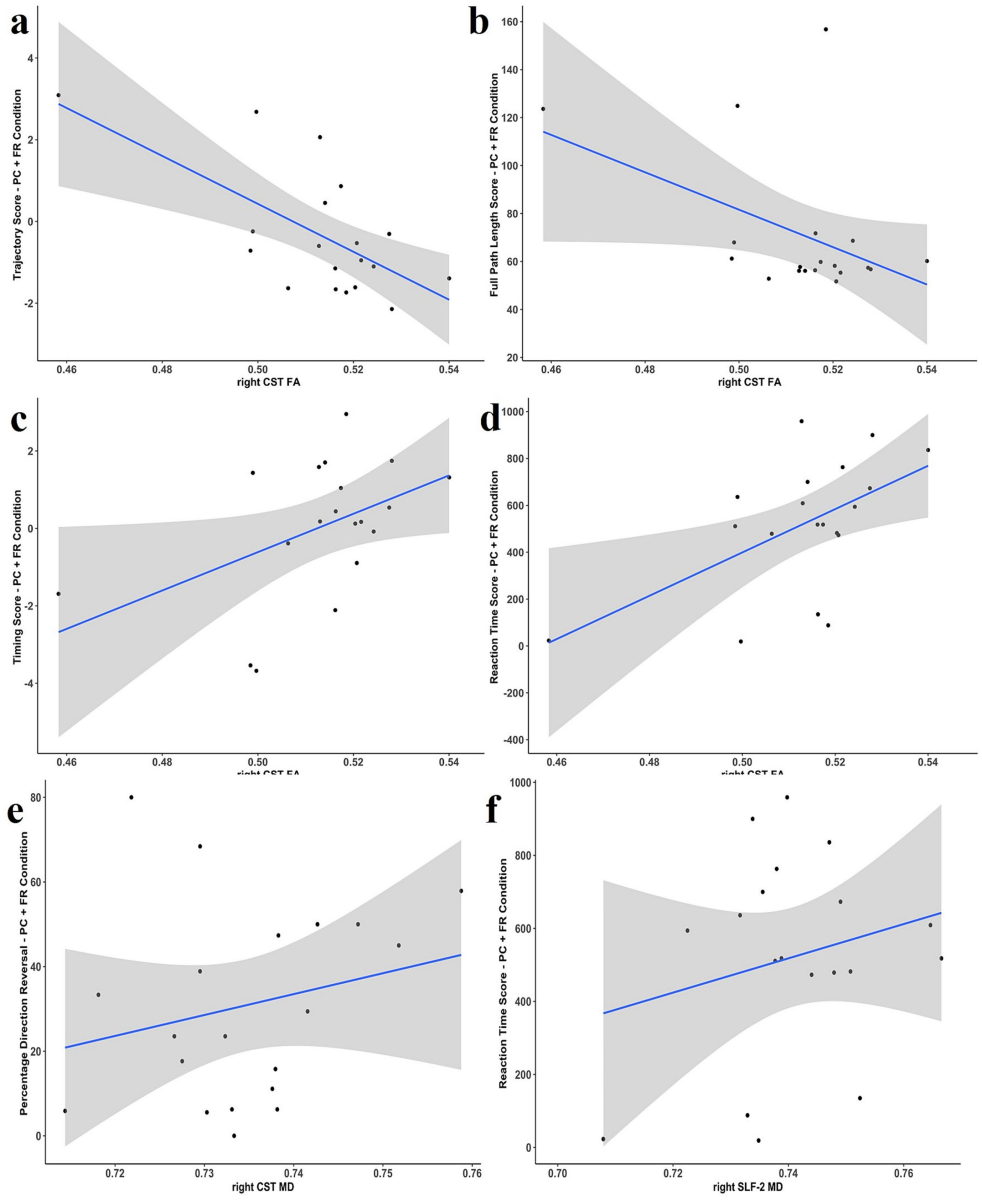
Relationship between FA in the entire-tract and PC + FR condition: **(a)** Right CST and trajectory score; **(b)** right CST and full path length score; **(c)** right CST and timing score; **(d)** right CST and reaction time score. Relationship between MD in the entire-tract and PC + FR condition: **(e)** Right CST and percentage directional reversal; **(f)** right SLF-2 and reaction time score. CST, corticospinal tract; FA, fractional anisotropy PC + FR, plane change and feedback reversal. CST, corticospinal tract; MD, mean diffusivity; PC + FR, plane change and feedback reversal; SLF-2, superior longitudinal fasciculus 2.

Similarly, results from multivariate multiple regression analysis revealed a positive association between RPQ-3 and the entire right SLF-2 MD (Table 6; Figure 8d). We found no associations in DHI. In all tracts, an increase in MD denoted worse PPCS and dizziness- related symptoms. Additionally, the entire-tract analysis revealed various significant associations with multiple tracts (Table 6): right CST FA with trajectory score, PLf, timing score, and RT (Figures 9a–d); right CST MD with DR (Figure 9e); PLF with left ILF MD, left SLF-1 MD, and bilateral SLF-2 MD (Table 6); right SLF-2 MD with RT (Figure 9f).

No associations were observed either between severity of PPCS or dizziness-related symptoms and upper limb visuomotor performance.

## 4 Discussion

The main goal of this pilot study was to investigate the functional and structural neural underpinnings of visuomotor performance, and to examine the effects of PPCS and vestibular dysfunction on the observed neuroanatomical correlates. The primary findings were: (1) higher RSFC within the SMN, DAN, and FPCN were associated with worse PPCS outcomes; (2) lower inferior parietal thickness was related to worse vestibular dysfunction; (3) higher RSFC within SVAN was associated with better visuomotor performance, while lower superior parietal thickness was associated with worse visuomotor performance on the task requiring two levels of decoupling (PC + CR), and (4) evidence of significant associations between white matter integrity of entire tracts and along specific sections of the long-associative, projective, and commissural tracts with PPCS, vestibular dysfunction, and visuomotor performance. The alteration in white matter integrity was mostly observed on tracts implicated in visuomotor control, particular in association with the challenging CMI task.

Disruption in RSFC in working-aged adults with PPCS has previously been reported (So et al., 2023). We found evidence of increased connectivity in the SMN and DAN were related to early symptoms of PPCS (headache, dizziness, and nausea) although these symptoms can persist long after the injury, while increased connectivity in FPCNb was associated with later psychological and cognitive symptoms. Previous studies examining functional connectivity in TBI have reported both increased and decreased connectivity (Han et al., 2016; Konstantinou et al., 2019; So et al., 2023; Stevens et al., 2012; Wong et al., 2023). The mixed results might be attributed to factors including sample size, study design, age, sex ratio imbalance, recovery phase, among others. Our findings are in accordance with some studies that reported increased RSFC among these networks (Champagne et al., 2020; Mayer et al., 2011, 2015; Simos et al., 2023; So et al., 2023). Wong et al. (2023) observed increased RSFC within the DMN and executive central network/FPCN were associated with improved neurocognitive performance in working-aged females with PPCS. Another study reported an increase in RSFC in multiple networks including the DAN, FPCN, and SMN were associated with decreased behavioral symptoms in individuals with mTBI (Amir et al., 2021). The FPCN consists of two distinct subnetworks (FPCNa and FPCNb) which are functionally connected to the DMN and DAN, respectively (Dixon et al., 2018; Murphy et al., 2020). The connectivity between FPCNb and DAN plays a key role in visuospatial perceptual attention and is required for encoding and maintaining task-relevant information in working memory (Dixon et al., 2018; Ptak, 2012; Ptak et al., 2017). Therefore, the observed increased connectivity in the current study may possibly indicate neuro-compensatory adaptive efforts, perhaps in form of functional reorganization near or distal to the site of injury (Rosenthal et al., 2018). Further research (including larger sample size, matched healthy controls and longitudinal follow-up) is warranted to substantive this claim.

Given the role of the SVAN in switching between networks (Menon and Uddin, 2010), and the FPCN’s role in externally-directed cognition (Dixon et al., 2018), our observed increased connectivity in the SVAN and shorter full path in the PC + FR condition may reflect a predisposition to recruiting additional cognitive resources when engaging in task(s) with increasing complexity in order to achieve a successful performance. Our results were further corroborated by the lack of significant relationship between the DMN and both visuomotor conditions, thus suggesting greater SVAN-FPCN connectivity and the involvement of SVAN in complex visuospatial attention and visuomotor task (Benassi et al., 2021; Diwakar et al., 2015; Ohashi et al., 2018; Vossel et al., 2014), particularly in rule-based CMI. Taken together, our RSFC findings suggest that the networks implicated in the integration of sensorimotor and cognitive-motor systems, particularly during the decoupling of visuomotor control, are especially susceptible to concussion.

Moreover, we observed several associations between measures of white and gray matter integrity with PPCS and visuomotor performance. Specifically, lower cortical thickness in the inferior parietal and superior parietal cortices were associated with worse vestibular dysfunction and poor performance on the PC + FR condition, respectively. Our findings are consistent with Zhe et al. (2021) who reported decreased cortical thickness in the superior parietal lobule and lower sulci depth in the inferior and superior parietal lobules in patients with vestibular migraine. Similarly, another study from our group demonstrated lower cortical thickness in the inferior and superior parietal lobules were associated with poor performance in various CMI-based tasks in participants with PPCS (Hurtubise et al., 2022). As part of the multisensory vestibular cortical network (Dieterich and Brandt, 2008), the inferior parietal cortex is involved in vestibular processing and responsible for sustained attention when engaging in ongoing tasks and responding to salient stimuli in space (Singh-Curry and Husain, 2009). Also, the superior parietal cortex is involved in sensorimotor integration and responsible for spatial orientation using proprioceptive cues during reaching movements (Sabes, 2000). Thus, given the contribution of these posterior parietal cortices in sensorimotor transformations between visual inputs and motor outputs and spatial awareness, it is unsurprising that structural changes can result in poor hand-eye coordination and balance, as demonstrated in the current study.

We found that higher MD along the CC splenium and in the whole SLF-2 were associated with worse PPCS, while higher MD along the CC splenium and premotor were associated with increased vestibular dysfunction. Our findings reinforce results from previous studies with different sample sizes and cohorts that have reported diffused white-matter damage linked to persistent symptoms after concussion (Gumus et al., 2022; Leh et al., 2017; Multani et al., 2016; Murdaugh et al., 2018; Mustafi et al., 2022; Rabinowitz et al., 2014; Taghdiri et al., 2018). Calzolari et al. (2021) showed decreased FA and increased MD in the right ILF was associated with impaired balance in TBI patients with vestibular agnosia, contrary to our vestibular findings. However, another study from the same group demonstrated that vestibular agnosia was associated with increased RSFC between the SLF and the posterior corona radiata tracts (Hadi et al., 2022), supporting our findings. The SLF is an associative multi-sectional tract functionally connecting the frontoparietal control network and the posterior parietal lobule anatomically, and the integrity of the SLF-2 is related to visuospatial and visuomotor control processes (Janelle et al., 2022; Nakajima et al., 2020), whereas the CC splenium and CC-body premotor serve as connections between the parieto-occipital and motor areas, respectively (Maffei et al., 2021; Park et al., 2008). Together, these tracts are critical for various top-down cognitive and vestibular sensorimotor processes that are vulnerable to the shearing and tearing forces due to concussion (Gumus et al., 2021; Narayana, 2017; Ubukata et al., 2016). Therefore, emphasizing the importance of investigating the microstructural diffusion changes along white matter tracts.

In the present study we observed an overall association between FA, MD, and visuomotor performance across both conditions. Our finding supports prior results from our group and others that implicates healthy white matter integrity for successful CMI task performance (Gorbet and Sergio, 2016; Hawkins et al., 2015; Hurtubise et al., 2020; Rogojin et al., 2023). The whole and along-tract analyses between visuomotor performance scores and DTI measures in several long-coursing and projection tracts (ILF, SLF-1 and 2, and CST) (see Tables 5, 6) showed that alterations in white matter integrity (increased and/or decreased FA and MD) were associated with increased movement errors, decreased movement accuracy, and slowed psychomotor response. Conflicting results from studies examining concussion-related white matter integrity changes may arise from multiple factors like differences in imaging and methodology protocols, heterogeneity in injury severity and participants, variability in injury mechanism, and injury phase, with some reporting inconsistent changes in FA and MD directions. For instance, some studies have reported decreased FA and increased MD during the acute and chronic phases (Kraus et al., 2007; Nakayama, 2006; Niogi et al., 2008; Veeramuthu et al., 2015), while others have reported increased FA and decreased MD in both phases (Bazarian et al., 2007; Henry et al., 2011; Veeramuthu et al., 2015; Wilde et al., 2012). Furthermore in concussion, vasogenic edema is characterized by reduced FA and increased MD because of the release of intracellular protein into brain parenchyma and may be reversible, while cytotoxic edema is related to increased FA and decreased MD due to the abnormal accumulation of intracellular fluid and cell swelling and may be irreversible (Michinaga and Koyama, 2015). These pathogenic edematous processes coupled with demyelination could explain the visuomotor impairment observed in the current study months or even years post initial injury. Consistent with our results, Hurtubise et al. (2020) found worse performance on the non-standard (PC + FR) condition indicated by lower trajectory composite score was related to decreased FA in the ILF, SLF, and CST despite the lack of associations between PPCS, standard visuomotor condition, and white matter MD. Similarly, two studies reported lower FA and higher MD, AD, and RD in ILF, SLF, cingulum, CST, CC, and forceps tracts predicted impaired performance in the PC + FR condition in APOEe4 carriers (Hawkins et al., 2015; Rogojin et al., 2023). As previously mentioned, these white matter tracts pass through the task-positive networks, connecting regions such as the posterior parietal lobule that are involved in visuomotor processing. Furthermore, our CMI condition involved two levels of decoupling that required both implicit sensorimotor recalibration and the explicit strategic control. Such task complexity putatively requires intact large-scale functional and structural network integrity. Hence, disruption of the normal homeostatic neural state and abnormal changes in structural components, combined with the already taxed metabolic capacity required for concussion recovery, may place an energetic strain on the control system resulting in poor motor performance (Bigler, 2013; Hillary and Grafman, 2017).

### 4.1 Limitations, future directions, and strengths

Our study has both limitations and strengths. Firstly, since this was not a longitudinal study, we could not address the causal relationships between RSFC, abnormal white matter integrity, lower cortical thickness, and impaired visuomotor performance in working-aged adults with PPCS. Future studies should investigate the long- term interactive effects of functional and structural integrity on developing PPCS and visuomotor deficits in working-aged adults. Secondly, the absence of informant verified self-reported PPCS and number of concussions may have introduced bias. Thirdly, the lack of a control group meant that we could not compare the performance on visuomotor tasks between participants with PPCS and healthy controls and we could not determine the specific neuroanatomical correlates of PPCS. Since, neuroimaging studies in healthy controls are crucial in identifying the functional and structural neural underpinnings of visuomotor performance, their absence in our study might impact the generalization of our findings. Moreover, the interpretation and generalizability of our findings is limited by our small sample size. Therefore, more studies with healthy controls and a larger sample size are required to confirm our results and further investigate the effects of these variables on PPCS, brain function and structure, and visuomotor performance in working-aged adults. Our sample contained proportionately more females, which was consistent and contrary to some TBI studies (Biegon, 2021; Shafi et al., 2020; Smeha et al., 2022; So et al., 2023). As a result, we were unable to explore any sex differences in PPCS and its potential associations with functional and structural integrity and visuomotor performance. Potential sex differences may result from multiple factors including sex hormones, PPCS profiles, length of recovery, neuroanatomy and function, neck muscle strength, and head/neck ratios (Chaychi et al., 2022; McGlade et al., 2015; Shafi et al., 2020; Tiernery et al., 2005; Varriano et al., 2018). Hence, future work ongoing in our laboratory will expand on previous research that have observed sex differences in visuomotor performance in working-aged with PPCS (Smeha et al., 2022) by incorporating the aforementioned factors that may explain possible mechanisms underlying sex differences in concussion.

A main strength of our study was the inclusion of working-aged adults with various concussion mechanisms. Prior studies investigating the impact of concussion and PPCS on brain health and quality of life have typically examined elite athletes thereby, excluding participants from the community with various exercise levels and severity of PPCS and concussion. The inclusion of these participants in our study strengthens the generalizability of our findings beyond sport-related concussion and elite athletes. An additional strength was the implementation of along- tract DTI profile analysis to examine the relationships between functional and structural integrity and visuomotor performance in participants with PPCS. The approach was imperative because it provided an opportunity to determine any focal microstructural changes along each white matter tract that may be missed when averaging values over the whole tract.

### 4.2 Conclusion

Our findings suggest that visuomotor tasks may have the potential for detecting neural changes related to functional and structural integrity in individuals with PPCS. We showed that persistent symptoms after concussion are associated with increased connectivity within several functional networks. Specifically, we observed increased connectivity within the salience ventral attention network was associated with better performance on a challenging visuomotor task that required cognitive-motor integration. Additionally, overall visuomotor deficits and more severe persistent symptoms with vestibular dysfunction were associated with lower cortical thickness in the posterior parietal lobule and decreases in white matter integrity, especially along the long-association, projection, and commissural tracts. Future longitudinal research may benefit from investigating possible mechanisms underlying the neuro-compensatory efforts in brain recovery and how to extend the observed mechanisms, especially in the presence of diminishing structural integrity. These interventions may help in managing persistent symptoms after concussion that can contribute to impaired visuomotor performance, particularly on activities that require complex integration of sensorimotor and cognitive-motor systems.

## Data Availability

The raw data supporting the conclusions of this article will be made available by the authors, without undue reservation.

## Glossary

AD: Axial diffusivity
AE: Absolute error
AFNI: Analysis of functional neuroimages
ANTs: Advanced normalization tool
BOLD: Blood oxygen level-dependent
CC: Corpus callosum
CI: Confidence intervals
CMI: Cognitive-motor integration
CST: Corticospinal tract
DAN: Dorsal attention network
DHI: Dizziness-related inventory
DMN: Default mode network
DR: Directional reversal
DSM − 5-TR: Diagnostic and statistical manual of mental disorders
DTI: Diffusion tensor imaging
DWI: Diffusion weighted images
EPI: Echo planar imaging
FA: Fractional anisotropy
fMRI: Functional magnetic resonance imaging
FOV: Field of view
FPCN: Frontoparietal control network
FPCNa: Frontoparietal control network part a
FPCNb: Frontoparietal control network part b
FPCNc: frontoparietal control network part c
FWHM: Full-width half-maximum
GLM: General linear model
GPIP: Group prior individual parcellation
ICs: Independent components
ICD-11: International Classification of Diseases Version 11
ILF: Inferior longitudinal fasciculus
LOC: Loss of consciousness
MCP: Middle cerebellar peduncle
MD: Mean diffusivity
ME-ICA: Multi-echo independent components analysis
MP-RAGE: Magnetization-prepared rapid gradient echo
mTBI: Mild traumatic brain injury
MTf: Full movement time
PASTA: Pointwise assessment of streamline tractography attributes
PC + FR: Plane change + feedback reversal
PPCS: Persistent post-concussion symptoms
PL: Path length
PLb: Full path ballistic
PLf: Full path length
PV: Peak velocity
RD: Radial diffusivity
ROI: Region of interest
RPQ: Rivermead post-concussion Symptoms questionnaire
RPQ-13: Rivermead post-concussion Symptoms questionnaire group 2
RPQ-3: Rivermead post-concussion Symptoms questionnaire group 1
RSFC: Resting state functional connectivity
rsfMRI: Resting state fMRI
RT: Reaction time
SD: Standard deviation
SLF: Superior longitudinal fasciculus
SLF-1: Superior longitudinal fasciculus part 1
SLF-2: Superior longitudinal fasciculus part 2
SMN: Sensorimotor control network
sMRI: Structural MRI
SVAN: Salience ventral attention network
TE: Echo time
TR: Repetition time
TRACULA: TRActs Constrained by UnderLying Anatomy
VE: Variable error
VN: Visual network
